# Social connectedness and negative affect uniquely explain individual differences in response to emotional ambiguity

**DOI:** 10.1038/s41598-020-80471-2

**Published:** 2021-02-16

**Authors:** Maital Neta, Rebecca L. Brock

**Affiliations:** Department of Psychology, University of Nebraska-Lincoln, Lincoln, USA

**Keywords:** Human behaviour, Emotion

## Abstract

Negativity bias is not only central to mood and anxiety disorders, but can powerfully impact our decision-making across domains (e.g., financial, medical, social). This project builds on previous work examining negativity bias using dual-valence ambiguity. Specifically, although some facial expressions have a relatively clear negative (angry) or positive valence (happy), surprised expressions are interpreted negatively by some and positively by others, providing insight into one’s valence bias. Here, we examine putative sources of variability that distinguish individuals with a more negative versus positive valence bias using structural equation modeling. Our model reveals that one’s propensity toward negativity (operationalized as temperamental negative affect and internalizing symptomology) predicts valence bias particularly in older adulthood when a more positive bias is generally expected. Further, variability in social connectedness (a propensity to seek out social connections, use those connections to regulate one’s own emotions, and be empathic) emerges as a notable and unique predictor of valence bias, likely because these traits help to override an initial, default negativity. We argue that this task represents an important approach to examining variability in affective bias, and can be specifically useful across the lifespan and in populations with internalizing disorders or even subclinical symptomology.

## Introduction

Negativity bias, which can be conceptualized as an enhanced attention to and memory for negative emotional information^1–3^ and a tendency to interpret ambiguous information as negative, has been thought to underpin the development and persistence of mood and anxiety conditions^4,5^. For example, in an otherwise healthy population, those with higher negativity bias show greater depression^6^, and altered emotional brain function compared to those with a positivity bias^7,8^. Such a bias could be related to exaggerated emotional responses and/or a failure of regulatory control to suppress distracting emotional influences^9^. Indeed, some work has shown that this bias can be overridden with emotion regulation strategies^10^.

While mood and anxiety disorders are both widespread and debilitating, there are important gaps in the literature linking them with negativity bias (see^11^). For example, much of the work on negativity bias does not allow for measuring a wide range of individual differences in negativity or provide much of an opportunity to override the negativity. Indeed, extant literature in depression and anxiety^2,12^^,^^13^, including subclinical symptomology^14^, has focused on responses to clear valence (e.g., sad or angry expressions), and described exaggerated responses to negativity as an *attentional* bias (toward negativity and away from positivity; see also^15^, which may suffer from floor or ceiling effects. In other words, when the valid response options are skewed to one end of the valence spectrum (neutral to negative or neutral to positive), rather than providing valid response alternatives ranging from negative to positive, the response variability is necessarily limited.

Even outside of work examining clear valence, the majority of work with ambiguity has focused on responses that are skewed to one end of the valence spectrum. For example, some research has examined responses to stimuli where the alternate meanings were only negative or neutral (e.g., “die”; i.e., there was not an equally valid positive interpretation^16^, but see Joorman et al.^17^), and found that individuals with a negativity bias show a stronger propensity toward the negative or threatening interpretation (see related work using response alternatives that are positive or neutral, e.g.,^18^. In still other studies leveraging ambiguity, the stimulus ambiguity was created in the laboratory by morphing different facial expressions (i.e., weakening its ecological validity) and measuring one’s tendency to perceive negative emotions (e.g., anger, fear, disgust) in the morphed expressions^19,20^.

In contrast, our work represents a methodological advance that builds on the concept of negativity bias using ecologically valid, dual-valence ambiguity. For example, although some facial expressions have a relatively clear negative (e.g., angry) or positive valence (e.g., happy), other expressions (e.g., surprise) are ambiguous because they signal both negative (e.g., witnessing an accident) and positive events (e.g., receiving an unexpected gift). When presented without contextual information, these ambiguous expressions are used to identify one’s valence bias, or the tendency to interpret ambiguity as having a more negative or positive meaning (e.g.,^21–23^). In this paradigm, the valence bias (which is conceptually similar but distinct from the construct of negativity bias) is not related to attention allocation—both positive and negative information are available within the visual information provided. Rather, it measures one’s tendency to perceive negativity when both negative and positive response options are equally valid. As such, this task does not constrain the valence of the participant’s response. Further, in contrast to neutral faces that are devoid of emotional meaning (i.e., neither negative or positive response are necessarily valid), surprise has a meaning (high arousal) that predicts both positive and negative outcomes (dual-valence representation) depending on the context. Thus, our stimuli allow us not only to determine the propensity of individuals to find positive meaning in ambiguity but is being used in manipulations that train them to do so^24^.

Extending beyond the context of negativity bias and affective disorders, valence bias can also powerfully impact our decision-making across many domains. Indeed, decision-making under uncertainty is ubiquitous in daily life (e.g., financial, medical, and social decision-making; see^25–27^ for respective reviews) and is particularly salient in the current worldwide pandemic (e.g.,^28,29^). Thus, our responses to uncertainty can have dramatic and widespread consequences. For instance, a more negative bias in response to uncertainty might preclude individuals from reaping benefits associated with potential rewards (e.g., stock market gains, lottery winnings). On the other hand, a more positive bias exposes individuals to aversive outcomes (e.g., economic downturns, financial loss). Similarly, uncertainty may arise when judging another’s trustworthiness^30^, or gauging the thoughts of others^31^, and the bias evident when resolving this uncertainty can facilitate or obstruct social affiliation and even enflame intergroup conflict (e.g.,^32,33^).

Despite the far-reaching implications of valence bias, there is very little research that explores the underpinnings of this bias. Given that differences in valence bias are stable (i.e., high test–retest reliability across several months: *r* = 0.46, *p* < 0.001; unpublished findings; and also across 1 year in a smaller sample: *r* = 0.72, *p* < 0.001^23^, and they are likely to have important consequences for social and emotional functioning, including mental health (depression/anxiety), the present research was directed at exploring the multiple putative sources of variability that might distinguish individuals with a negative valence bias from those with a more positive bias. Indeed, there is extensive evidence suggesting that trait differences in personality are associated with variability in behavioral and neural responses to emotion^34,35^. For example, it is well-established that neuroticism explains variability in negativity bias^36–39^ and is related to mood and anxiety symptoms in both clinical^40^ and nonclinical samples^41^. And emerging research provides some evidence to suggest that valence bias per se is related to trait-like differences, including trait anxiety^42^. Thus, we might expect that neuroticism, and other related forms of trait and state negative affectivity already discussed (e.g., anxiety, depression, emotion dysregulation), may provide important insight into the tendency toward a more negative valence bias.

Interestingly, we have demonstrated, through of a variety of approaches, that the initial or default interpretation of ambiguous (surprised) faces is negative^23,43–46^, and the positive interpretations require an additional regulatory process that overrides this initial negativity^47^. As such, in addition to the variability associated with negative affect, there are likely to be trait differences that promote a more positive valence bias that help to override the initial negativity bias. For example, extraversion is associated with greater positive affect^36^ and decreased mood and anxiety symptoms in both clinical^40^ and nonclinical samples^41^. In addition, studies of valence bias per se have demonstrated that greater empathy is associated with a more positive bias^23^. Given the initial negativity, it could be that individual differences associated with social connectedness (one’s propensity to seek out social connections, use those connections to regulate one’s own emotions, and be empathic) are even more important in explaining valence bias than is variability associated with negativity. Notably, there is an important link between social connectedness (often operationalized in the literature as the quantity and/or quality of social ties) not only with increased positive emotions and well-being^48–50^ and increased empathy^51^, but also with ameliorating symptoms of disorders characterized by a negativity bias (i.e., depression, anxiety^52–55^, across the lifespan^56^.

### The present approach

In the present study, we set out to (a) determine the putative sources of variability that might distinguish individuals with a more negative versus positive valence bias, and (b) establish the importance of the valence bias task in exploring individual differences relevant to subclinical symptomology. We conducted an analysis of data collected across 14 experiments that similarly assessed valence bias in each participant, and also collected a variety of individual difference measures thought to have a potential role in explaining valence bias. Our hypothesis is that valence bias is related to (some set of) individual differences in measures of negative affectivity and social connectedness. We predict that the effect of social connectedness will be particularly important in promoting a positive valence bias given that, at least in young adults, the negative bias represents the initial or default response. In other words, although there are likely important individual differences that reinforce a more negative bias, the ability to override the default negativity may more critically rely on variability in social connectedness that helps to downregulate or overcome the tendency toward negativity. In contrast, a low propensity toward experiencing negative emotions (low sensitivity to distressing stimuli) may not be sufficient to demonstrate a positive bias, at least in young adulthood.

## Results

### Valence bias

**Figure 1:**
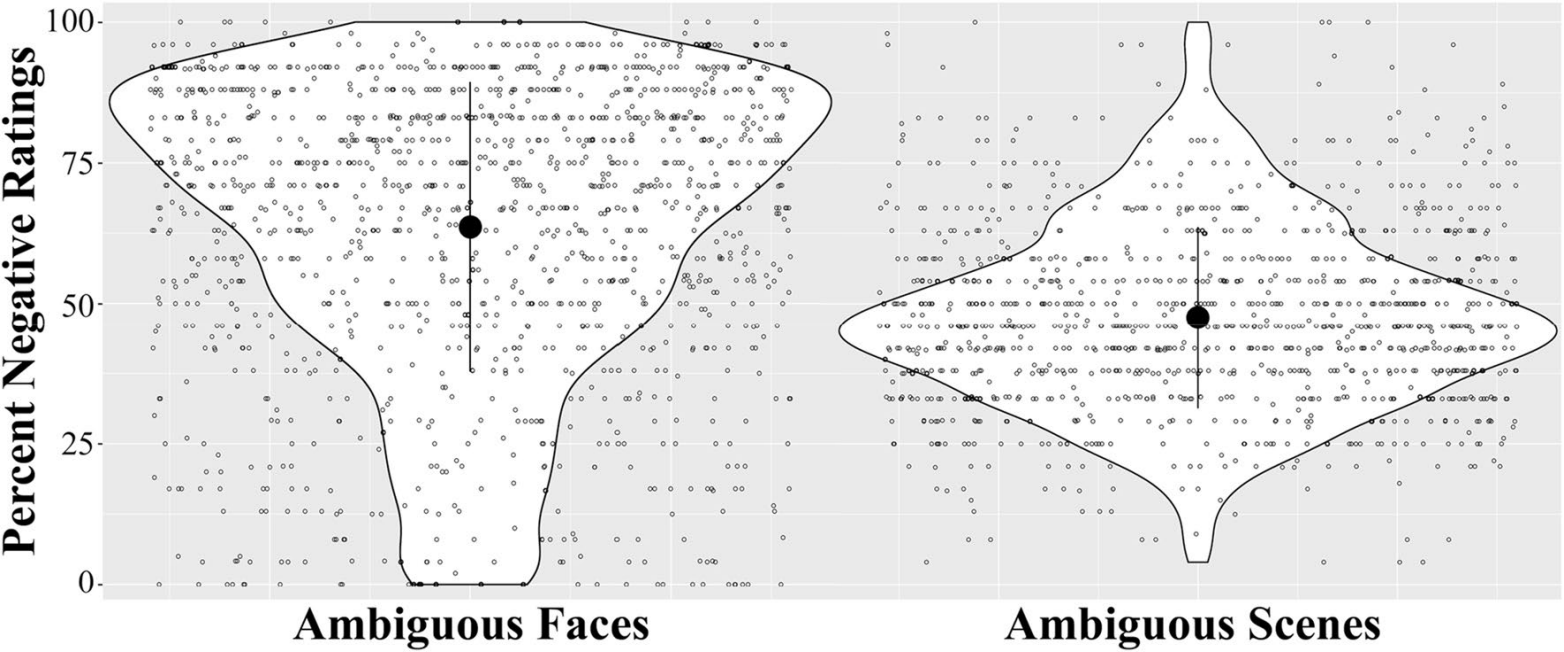
Distribution of valence bias (operationalized as percent negative ratings) in response to ambiguous (surprised) faces and scenes. The central dot represents the mean, and whiskers represent standard deviation; each participant is plotted with an open circle.

As in previous work, there was a wide range of inter-participant variability in valence bias for both the faces and scenes (see Fig. 1; higher scores are associated with a more negative bias). Interestingly, the ratings of ambiguous faces were more negative and also more variable across participants than ratings of scenes. As expected, valence bias across stimuli was significantly positively correlated, *r* = 0.22 (see Table 1); however, the correlation was small in magnitude.

**Table 1:** Correlations among dependent variables.

Variable	1	2	3	4	5	6	7	8	9	10	11
1. BDI											
2. DERS	0.53***										
3. NEON	0.66***	0.60***									
4. STAIS	0.60***	0.53***	0.62***								
5. STAIT	0.78***	0.62***	0.79***	0.78***							
6. EQ	− 0.15***	− 0.42***	− 0.25***	− 0.31***	− 0.31***						
7. IRQ	− 0.06*	− 0.13***	− 0.05*	− 0.16***	− 0.14***	0.36***					
8. NEOE	− 0.25***	− 0.39***	− 0.41***	− 0.38***	− 0.41***	0.49***	0.49***				
9. VB (Faces)	0.05	− 0.006	0.14***	0.08**	0.09***	− 0.008	− 0.05*	− 0.15***			
10. VB (Scenes)	0.06*	0.04	0.10***	0.05*	0.07**	0.01***	− 0.14***	− 0.06**	0.22***		
11. VB (Average)	0.07**	0.01	0.16***	0.09***	0.11***	0.04	− 0.11***	− 0.15***	0.87***	0.66***	
12. Age	− 0.20***	0.11***	− 0.23***	− 0.06*	− 0.17***	− 0.11***	0.05	− 0.14***	− 0.15***	− 0.16***	− 0.18***

***p < 0.001 (two-tailed), **p < 0.01 (two-tailed), *p < 0.05 (two-tailed).

*BDI* Beck Depression Inventory, *DERS* Difficulties in Emotion Regulation Scale, *NEON* neuroticism, *STAIS* state anxiety, *STAIT*-Trait Anxiety, *EQ* Empathy Quotient, *IRQ* Interpersonal Regulation Questionnaire, *NEOE* Extraversion, *VB* Valence Bias-higher scores associated with a more negative bias.

### Associations among measures

Correlations among concurrent measures were all in the expected direction (Table 1). The strongest association among indicators of negative affect was between neuroticism (NEON) and trait anxiety (STAIT; *r* = 0.79), and the weakest was between depression symptoms (BDI) and difficulties in emotion regulation (DERS; *r* = 0.53). The strongest association among indicators of social connectedness was between empathy (EQ) and extraversion (NEOE; *r* = 0.49), and the weakest was between empathy (EQ) and interpersonal emotion regulation (IRQ; *r* = 0.36). As expected, average valence bias (i.e., higher values represent a more negative bias) was significantly positively correlated with depression symptoms (*r* = 0.07), neuroticism (*r* = 0.16), and state (*r* = 0.09) and trait (*r* = 0.11) anxiety, and negatively correlated with interpersonal emotion regulation (*r* = − 0.11), extraversion (*r* = − 0.15) and age (*r* = − 0.18).

### Results of structural equation modeling

A measurement model with the three latent variables covarying with one another demonstrated adequate global fit (CFI = 0.938, TLI = 0.913, RMSEA = 0.066, 90% confidence interval = 0.058-0.074, SRMR = 0.057). The model explained a significant percentage of the variance in each indicator of negative affect: 66.5% for BDI (factor loading = 0.82), 93.0% for STAIT (factor loading = 0.97), 64.9% for STAIS (factor loading = 0.81), 68.1% for NEON (factor loading = 0.83), and 55.9% for DERS (factor loading = 0.75). The model explained a significant percentage of the variance in each indicator of social connectedness: 27.9% for EQ (factor loading = 0.53), 24.4% for IRQ (factor loading = 0.49), and 81.2% for NEOE (factor loading = 0.90). The model explained a significant proportion of variance in the two indicators of valence bias: 23.7% for faces (factor loading = 0.49) and 17.5% for scenes (factor loading = 0.42).

The three factors demonstrated adequate discriminant validity; negative affect was inversely correlated with social connectedness (*r* = − 0.52, *p* < 0.001) and positively with a more negative valence bias (*r* = 0.19, *p* < 0.001), and social connectedness was inversely correlated with a more negative valence bias (*r* = − 0.25, *p* < 0.001). We also examined correlations between the three latent factors and age which were significant (see Table 2). Interestingly, although there was a strong inverse correlation between negative affect and social connectedness, both were also inversely correlated with age. As predicted, age was also inversely correlated with a more negative valence bias (i.e., older adults had a more positive valence bias).

**Table 2:** Correlations among latent variables and age.

Variable	1	2	3
1. Negative affect			
2. Social connectedness	− 0.52***		
3. Valence bias	0.19***	− 0.25***	
4. Age	− 0.17***	− 0.13***	− 0.17***

***p < 0.001 (two-tailed), **p < 0.01 (two-tailed), *p < 0.05 (two-tailed).

After establishing adequate fit of the measurement model, we tested the full hypothesized model with negative affect, social connectedness, and age (z scored for interpretation) as predictors of valence bias, but omitted the interaction between negative affect and age. This step is necessary in the context of latent moderated structural equation models which do not produce traditional model fit indices. Maslowsky et al.^57^ recommend evaluating the global fit of the model without the interaction first, and then adding the interaction to examine its significance. The global fit of this model was adequate (CFI = 0.931, TLI = 0.903, RMSEA = 0.066, 90% confidence interval = 0.058-0.073, SRMR = 0.054).

**Figure 2:**
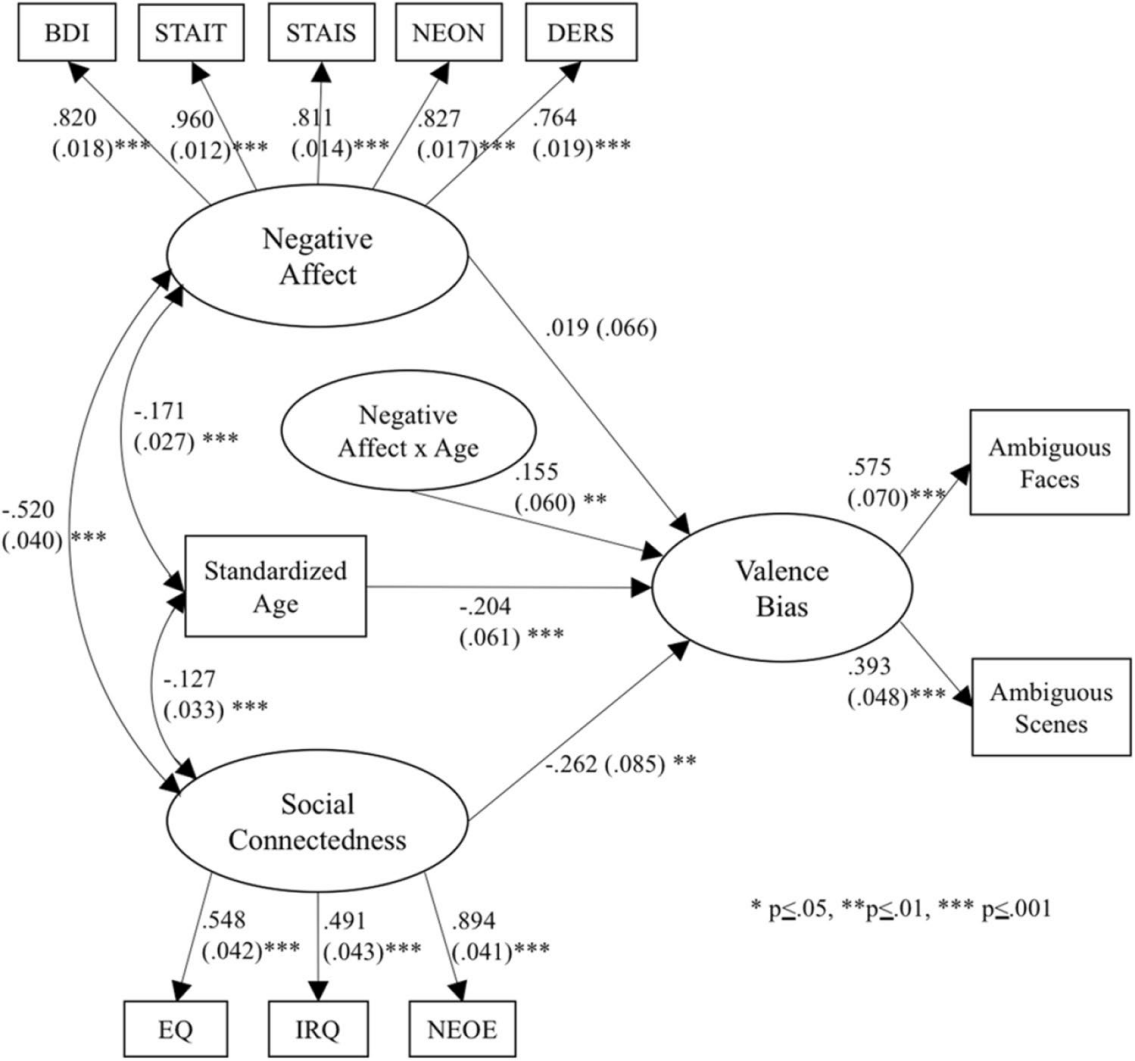
Results of the final model. Social connectedness was inversely associated with a more negative valence bias when controlling for negative affect and age. There was also evidence for a significant interaction between negative affect and age predicting valence bias, such that there was a stronger positive association between negative affect and a more negative bias for older individuals. Values along each arrow are standardized estimates, with associated standard error in parentheses, and significance level represented by asterisks. This model explained 12.9% of the variance in Valence Bias. *BDI* Beck Depression Inventory, *DERS* Difficulties in Emotion Regulation Scale, *NEON* Neuroticism, *STAIS* State Anxiety, *STAIT* Trait Anxiety, *EQ* Empathy Quotient, *IRQ* Interpersonal Regulation Questionnaire, *NEOE* Extraversion.

Next, we added the latent interaction between negative affect and age to the model which was significant, b = 0.17 (0.06), *p* = 0.010. Note that because there were 113 participants who had missing scores on both variables used to define the latent interaction (i.e., negative affect and age), those cases were not included in this model. Figure 2 shows the final model results (see Supplementary Table S3 for the unstandardized model solution). As expected, social connectedness was inversely associated with a more negative valence bias when controlling for negative affect and age, β = − 0.26, *p* = 0.002. There was also a significant interaction between negative affect and age predicting valence bias, β = 0.16, *p* = 0.01, such that there was a stronger positive association between negative affect and a more negative valence bias for older individuals.

**Figure 3:**
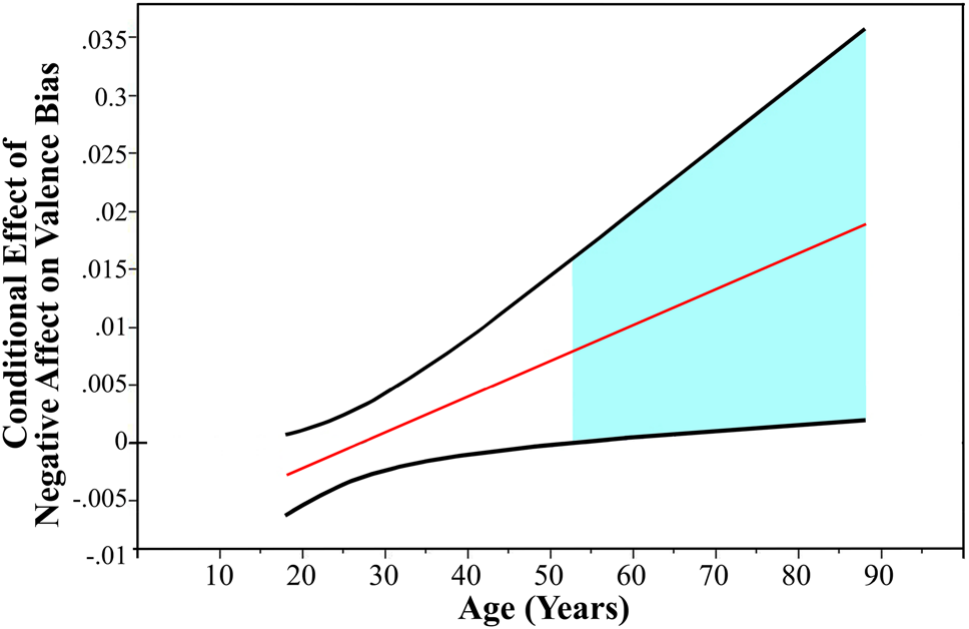
Depiction of the significant interaction between negative affect and age. The straight line represents conditional effects of negative affect on valence bias at different ages and the curved lines reflect the 95% confidence interval for those effects. Negative affect was positively associated with a more negative valence bias, controlling for social connectedness, for individuals age 51.6 and older (i.e., beginning at 1.95 SDs above the mean of age).

To probe the significant interaction, we conducted a regions of significance analysis such that conditional effects of negative affect on valence bias were estimated at all observed levels of age, and the significance of those conditional effects were examined. Negative affect was positively associated with a more negative valence bias, controlling for social connectedness, for individuals age 51.6 and older [i.e., beginning at 1.95 SDs above the mean of age, M (SD) = 28.06 (12.07); Fig. 3].

Finally, based on some work showing that older age is associated with a greater investment in more rewarding and meaningful social relationships^58^, we explored a supplementary model that included the latent interaction between social connectedness and age, but this interaction was not significant, b = − 0.14 (0.09), *p* = 0.094.

## Discussion

There are vast individual differences in the tendency to interpret dual-valence ambiguity as having a more positive or negative meaning. This valence bias is not only central to mood and anxiety disorders, but can powerfully impact our decision-making across domains (e.g., financial, medical, social), and thus have dramatic and widespread consequences for many aspects of our lives. To examine the underlying sources of variability that might distinguish individuals with a more negative versus positive valence bias, we conducted an analysis across fourteen experiments. Broadly, we found that both negative affect and social connectedness appear to *uniquely* explain individual differences in valence bias. This finding is consistent with extensive research that has demonstrated an important link between negativity bias and mood and anxiety disorders^1–3^. We have built on this link by demonstrating a relationship between valence bias and negative affect, which was conceptualized as a latent measure representing temperamental negative affect and internalizing symptomology. Although negative affect was significantly correlated with a more negative valence bias (Table 2), upon examining its unique association with bias (i.e., controlling for social connectedness), it was only associated with valence bias for older adults. In other words, older adults (i.e., around 51 years of age and up) that were higher in negative affect showed a more negative valence bias independent from social connectedness (i.e., no such association was evident in younger adults).

Interestingly, prior work on valence bias in younger adults has demonstrated that the initial or default interpretation of ambiguity is negative^23,43–45^, and that positive interpretations require an additional regulatory process that overrides this initial negativity^47^. Other related work has shown that children show a more negative bias than adults putatively because regulatory mechanisms responsible for producing a positive bias are weaker in children than adults^59^, see also^60^. As such, it could be that, while a negativity bias is associated with dysfunction (depression, anxiety), it is perhaps the relative failure to develop *mechanisms for regulating or overriding the negativity* that may serve both to maintain the negativity bias into adulthood and to increase the risk for disorders (see^61^).

Although there is an overall association between negative affect and valence bias in the current work, we found that negative affect was not uniquely predictive of valence bias after controlling for social connectedness in young adults. This finding might be due to their default negativity. In other words, when the default response is negative, the impact of negative affect (temperament and symptomology) may be diminished. In contrast, in older adults, a positive valence bias is more likely^43,62^ and may even represent the new default^63^. Thus, in this population, increases in negative affect appear to play a more crucial role in impacting valence bias. Alternatively, it could be that the interaction with age is due to some developmental process whereby, as individuals increasingly engage with more stimuli, there are more opportunities for their underlying proclivity toward negativity to manifest in a more negative valence bias, thus resulting in a more robust and stable bias. Future longitudinal work will be needed in order to disentangle these effects across different stages of the lifespan. Indeed, it could be that there are important mediators of the relationship between valence bias and negative affect (or negativity bias per se) in young adulthood (see^64^). Further, implementing this developmental framework would allow us to track within-person developmental processes and examine if and how this process strengthens over time.

Having said that, valence bias is measured along the full valence spectrum, from negative to positive; thus, we would be remiss to discuss the individual differences that support a negativity bias without mentioning the ramifications for positivity bias. We found that variability in social connectedness, which was conceptualized as a latent measure representing empathy, extraversion, and interpersonal emotion regulation (i.e., the tendency to rely on others in order to regulate one’s own emotions), was inversely associated with a more negative valence bias. In other words, individuals higher in social connectedness showed a more positive bias. Notably, although social connectedness is predominantly conceptualized as a facet of positive emotionality, low levels of social connectedness are significantly associated with greater negative affect (see Table 2). Results of the SEM analyses suggest that only the truly positive features of social connectedness (unique from negative affect) are predictive of valence bias.

These findings are consistent with extant work showing that extraversion is associated with greater positive affect^36^ and decreased negativity (i.e., decreased mood and anxiety symptomology^40,41^). Notably, our results are consistent with work showing that social connectedness increases positive emotions and well-being^48–50^ and mitigates internalizing symptoms^52–55^.

As briefly mentioned above, one might predict that variability in positive affect may be more important in predicting valence bias than the variability in negative affect, given that the negative bias represents the default response (in young adults). In other words, although there are important individual differences that reinforce a more negative bias, the ability to override the default negativity appears to more critically rely on variability in social connectedness that helps to downregulate or overcome the tendency to view dual-valence ambiguity in a negative light. In contrast, a low propensity toward experiencing negative emotions (low sensitivity to distressing stimuli) may be insufficient to demonstrate a positive bias, at least in young adulthood. Future longitudinal work could examine the development of these social connectedness measures over the course of one’s lifetime. This work could also prove useful in improving our ability to identify individuals who are at risk for maintaining this negativity bias and developing depression or anxiety (e.g., people low in social connectedness and therefore lower in positivity bias).

Given the theoretical connection between valence bias and negativity bias and its associated symptomology, we explored the bivariate associations between the average valence bias and individual measures within each latent construct (negative affect, social connectedness) and age. Consistent with our predictions, a more negative valence bias was positively correlated with four of the five individual measures within the negative affect construct: depression symptoms, neuroticism, and state and trait anxiety. Further, it was correlated with two of the three individual measures within the social connectedness construct: interpersonal emotion regulation and extraversion. It is intriguing that valence bias did not show a relationship with empathy, as in previous work^23^, future research will be needed to further explore this link.

As predicted, a more negative valence bias was inversely correlated with age. This finding is consistent with prior work demonstrating that increasing age is associated with a more positive valence bias^43,62^. Interestingly, although negative affect was inversely related to social connectedness, both of these constructs were inversely related to age (i.e., older adults showed decreases in negative affect and social connectedness). Future work targeting this aging population will be helpful to explicate the mechanisms underlying these changes, specifically as they relate to a shift toward a more positive valence bias.

In sum, although extant behavioral, psychophysiological, and neuroimaging research has provided important information about the valence bias, it has fallen short of elucidating the mechanisms underlying this variability. Extensive research has focused on dispositional negativity (see^65^ for a review), but here, we provide a model suggesting that the variance related to positivity (that may specifically help to overcome a negativity bias) is more sensitive to the valence bias than an approach that focuses on negativity. In other words, social connectedness emerged as a unique predictor of valence bias, and variability in negativity was an important predictor only in later life, when a positive bias is generally expected. In other words, a heightened sensitivity to subtle indicators of positivity and interpersonal connections might facilitate the override of default tendencies toward negativity, but a low propensity toward negative emotions (e.g., low depression/anxiety symptomology) is insufficient for a positive bias.

These results are consistent with previous work showing that specific traits are associated with the propensity to experience negative and positive affect, respectively^36^,^39^), and differences in neural responses to emotion^34,35^. However, the results build on existing research by establishing a methodological advance in studying valence bias that implements dual-valence ambiguity (both positive and negative information are present in the stimuli). These findings provide further evidence for the idea that this bias represents a relatively stable trait-like indicator of underlying individual differences^22,23^. As such, we argue that this task represents an important approach for future work examining variability in affective bias, and can be useful in research across age and in populations with related affective disorders or even subclinical symptomology.

Indeed, there are a variety of benefits of this performance-based measure of valence bias. First, although much of the literature on negativity bias has focused on comparing patients and controls (e.g.,^66^), our task has demonstrated a dimensional association between bias and subclinical depression symptomology^61^. Second, our task shows high test–retest reliability across one year, indicating that the bias is a trait-like difference across individuals^23^. It also generalizes across ratings of different stimuli, including ambiguously valenced (surprised) facial expressions, scenes, and emotionally laden words^22^,^67^. Third, it engages an amygdala-prefrontal cortex (PFC) circuitry^21,47,61^, similar to that implicated in disorders characterized by a negativity bias and emotion dysregulation^66,68^. Fourth, this task is developmentally appropriate—it has been leveraged for studying valence bias and its association with depressive symptomology and emotion regulation across the lifespan (ages 6–88 years), from children—including those experiencing early life stress^59–61^ to older adults^43,62,63^. Finally, it is sensitive to a range of contextual manipulations, including but not limited to stress^69^ and exercise^70^. As a result, this task offers a novel contribution to research on negativity bias using an approach that controls the information perceived by the participants (i.e., viewing the same dual-valence images) and enables a stable measure of bias.

Future work could also examine the functional outcomes of these effects. For example, given that differences in valence bias have important consequences for social and emotional function (e.g., depression/anxiety symptomology), further research should be directed at examining the utility of this model in distinguishing individuals with normal versus aberrant function. Results of this research can inform interventions by identifying individuals with heightened resilience or increased risk for affective disorders. Indeed, a positive valence bias is associated with resilience in the face of stress^71^. Further, ongoing work shows promise for emotion regulation training and mindfulness-based stress reduction see ^24^, both strategies used as interventions for mood and anxiety disorders, in promoting a more positive valence bias.

Given that social connectedness is a unique predictor of valence bias, and appears to support overriding the initial negativity, a specific focus on building social connections (e.g., training in interpersonal emotion regulation or loving kindness meditation; see^49^) might be explored as a putative intervention for ameliorating a chronic negativity bias.

## Methods

### Participants

Data from fourteen experiments, conducted on 1812 human participants at University of Nebraska-Lincoln and through Amazon’s Mechanical Turk (MTurk) were included in this analysis (Supplementary Table S1). A total of 326 participants were removed from the MTurk sample for the following reasons: 183 were removed because they failed to complete at least 75% of the experiment, an additional 104 were removed because they completed the experiment in less than 300 s (which was determined to not be feasible for the number of trials included in the study), four were removed because they did not complete the valence bias task, which was crucial for the analyses in the current report, and finally, 35 were removed for having inaccurate ratings in the valence bias task (we use a standard threshold of 60% accuracy as in previous work^23,43^). In addition, 96 participants that completed experiments in the lab were excluded: 64 for having inaccurate ratings in the valence bias task, and an additional 32 due to technical issues. The final sample resulted in 1390 adult participants (754 female, 562 male, 74 not reported). Age data was lost for 113 participants, but for the remaining sample, the age range was 17–88 years [mean (SD) age = 28.06 (12.07)]. Race data was lost for 73 participants, but for the remaining sample, there were 2 identifying as American Indian/Alaska Native, 117 as Asian, 61 as Black/African-American, 895 as White, 4 as Multiracial, 46 as Other, and 192 as Unknown/Choosing not to identify.

None of the participants were aware of the purpose of the experiment. All participants had normal or corrected-to-normal vision, and they were compensated for their participation through monetary payment or course credit. Before each session, written or electronic informed consent was obtained from all participants, with a waiver of informed consent from parents/legal guardians for minors that were enrolled as students at the University of Nebraska-Lincoln. All of the procedures were carried out in accordance with the relevant guidelines and regulations, and approved by the ethics committee of University of Nebraska-Lincoln for the Protection of Human Subjects. The only criteria used for selecting participants to include in the report was that they completed the valence bias task, and the same set of surveys, for use as the dependent variables in our analyses.

### Procedures

Across all experiments, participants first completed a valence bias task. In this task, images of faces from the NimStim^72^, Karolinska Directed Emotional Faces^73^ and Umea University Database of Facial Expressions^74^ sets and scenes from the International Affective Picture System (IAPS^75^), were presented. The faces included angry, happy, and surprised expressions, and the IAPS scenes were those previously identified as having a negative, positive, or ambiguous valence,ambiguity was defined in pilot work as those images that have a high standard deviation in valence ratings (i.e., individuals were in the most disagreement about their valence^22^). For both stimuli, previous work found that, unlike ratings of clearly negative and positive stimuli, there is a wide range of inter-participant variability in ratings of ambiguous faces and scenes^22,23^.

Participants viewed images of positive, negative and ambiguous images and rated (via keyboard press [n = 995] or mouse click [n = 395]) each image as either positive or negative. Response sides were counterbalanced across participants. Stimuli were presented for either 500 ms (n = 712) or longer (either 1000 ms or indefinitely as in the case for the Mturk studies; n = 678), depending on individual experiment settings. The number of blocks of trials varied slightly across experiments, but for all experiments, each block included presentation of the complete set of images (faces or IAPS, which were presented in separate blocks) presented in a pseudorandom order, and block order was counterbalanced between participants.

Task data were collected in several software packages: Eprime (Psychology Software Tools, Pittsburgh, PA, USA), MouseTracker^76^, and Qualtrics (Qualtrics, Provo, UT).

### Measures

#### Valence bias

The dependent measure used to quantify valence bias was percent negative ratings, which is calculated as the percent of trials on which a participant viewed an emotionally ambiguous stimulus and rated it as *negative*, out of the total number of trials for that condition (excluding omissions). Notably, previous work has demonstrated that the valence bias generalizes across faces and scenes; the same people that tend to rate surprised faces as positive also tend to rate ambiguous scenes as positive^22^. Given that these two valence bias scores should represent some stable, trait-like measure of the tendency to interpret ambiguity as having a more positive versus negative meaning, valence bias was represented by a latent construct using these two scores. This approach provides a more stable representation of valence bias that is common across the face and scene contexts. Having said that, to explore the bivariate correlations between valence bias and the other individual difference measures, we also created a bias score using the average of the face and scene ratings. This average score represents both the common and unique variance among the two measures. While most of the participants completed valence bias tasks with both stimuli, a subset of participants completed the task with only faces,for those latter individuals, the average valence bias score was treated as missing.

#### Negative affect

We administered a series of questionnaires to assess individual differences in negative affect. In order to account for missing data for any one question on a given questionnaire, which was minimal (< 1%), replacement scores were calculated with mean imputation by scale, and after reverse coding items as needed.

##### Depression symptomology

Scores were extracted from the Beck Depression Inventory-II (BDI^77^), one of the most widely used measures to assess severity of depression symptomology. This measure has demonstrated reliability and validity in a number of studies^77^. Each of the 21 items consists of four self‐evaluative statements, ranging in severity, for which respondents select the statement that best describes their symptoms from the last 2 weeks. Ratings from each item are summed, with a possible range of 0–63. In the present study, internal consistency was excellent (Cronbach’s alpha = 0.898).

##### Difficulty in emotion regulation

Scores were extracted from the Difficulties in Emotion Regulation Scale (DERS^78^) to assess typical levels of difficulties in emotion regulation. This measure is based on a clinically-useful conceptualization of emotion regulation that was designed to be applicable to a wide variety of psychological difficulties and relevant to treatment development for clinical populations^79^. The DERS is a 36-item, factor-analytically derived questionnaire that comprises six subscales representing unique dimensions of emotion regulation difficulties: (1) nonacceptance of emotional responses, (2) difficulty engaging in goal-directed behavior, (3) impulse control difficulties, (4) lack of emotional awareness, (5) limited access to emotion regulation strategies, and (6) lack of emotional clarity. The DERS has demonstrated excellent convergent and discriminant validity. Respondents rate how often each item applies to them using a 5‐point Likert scale (1 = *Almost Never*, 5 = *Almost Always*). Ratings from each item are summed to create a composite score across the six factors, with a possible range of 36–180. In the present study, internal consistency was excellent (Cronbach’s alpha = 0.948).

##### Neuroticism

Scores were extracted from the NEO Five-Factor Inventory (NEO–FFI^80^) to assess neuroticism (NEON). The NEO-FFI is one of the most widely used instruments to assess personality. This 60-item questionnaire includes scales to measure the big five personality traits, and respondents rate each item on the degree to which it is true or not true of them, using a 4‐point Likert scale (0 = *Strongly Agree*, 3 = *Strongly Disagree*). Due to differences in the survey procedure across studies, some participants only completed a subset of the 60-item questionnaire (e.g., neuroticism and extraversion scales only). Although the NEO-FFI has had multiple revisions since its original development in 1990, adequate reliability has been consistency demonstrated over the years^81^. The NEON scale consists of 12 items that were summed, with a possible range of 0–48. In the present study, internal consistency was excellent (Cronbach’s alpha = 0.862).

##### State and trait anxiety symptoms

Scores were extracted from the State-Trait Anxiety Inventory (STAI^82^) to assess state and trait anxiety symptomology. State Anxiety (STAIS) is measured with a 20-item questionnaire in which respondents rate each item on the degree to which it is true or not true of them, using a 4‐point Likert scale (1 = *Not At All*, 4 = *Very Much So*). Ratings from each item are summed, with a possible range of 20–80. Trait Anxiety (STAIT) is measured similarly, except respondents rate each item on the degree to which it is true or not true of how they “generally feel”. In the present study, internal consistency was excellent (Cronbach’s alpha: STAIS = 0.921, STAIT = 0.935).

#### Social connectedness

We administered a series of questionnaires to assess individual differences relevant to social connectedness. Again, mean imputation was used to account for any missing data on a given questionnaire (< 1%).

##### Empathy

Scores were extracted from the abridged Empathy Quotient (EQ^83^) to assess variability in empathy. The EQ is a relatively recent measure of empathy that is unique in that it was explicitly designed to have clinical application^84^. The abridged EQ includes 22 items that respondents rate on the degree to which it is true or not true of them, using a 4‐point Likert scale (2 = *Strongly Agree*, 1 = *Slightly Agree*, 0 = *Strongly* or *Slightly Disagree*). Ratings from each item are summed, with a possible range of 0–56. In the present study, internal consistency was excellent (Cronbach’s alpha: EQ = 0.888).

##### Interpersonal emotion regulation

Scores were extracted from the Interpersonal Regulation Questionnaire (IRQ^85^) to assess variability in one’s recruitment of social resources to up- and down-regulate one’s own emotions. This questionnaire includes measures of both tendency to recruit these social resources and efficacy with which these resources are perceived to be helpful, each with respect to managing both positive and negative emotions. The IRQ is a 16-item, factor-analytically derived questionnaire that is comprised of four subscales representing these unique dimensions: (1) negative emotions—tendency, (2) negative emotions—efficacy, (3) positive emotions—tendency, and (4) positive emotions—efficacy. The IRQ has demonstrated excellent convergent and discriminant validity, and is distinct from measures of negative expressivity, *intra*personal emotion regulation ability, and extraversion. Respondents rate each item on the degree to which it is true or not true of them, using a 7‐point Likert scale (1 = *Strongly Disagree*, 7 = *Strongly Agree*). Ratings from each item are summed to create a composite score across the four factors, with a possible range of 16–112. In the present study, internal consistency was excellent (Cronbach’s alpha = 0.932).

##### Extraversion

Scores were extracted from the NEO Five-Factor Inventory (NEO–FFI^80^) to assess extraversion (NEOE). As previously described, the NEO-FFI is one of the most widely used instruments to assess personality. The NEOE scale consists of 12 items that were summed, with a possible range of 0–48. In the present study, internal consistency was excellent (Cronbach’s alpha = 0.855).

#### Age and potential controls

A series of demographic characteristics (e.g., education, sex, household income, race, ethnicity, past history of mental or physical illness, medication use) as well as methodological differences across studies (i.e., presentation duration, number of trials, response method—mouse versus keyboard, study location—online versus laboratory) were examined. However, only age consistently correlated with both measures of valence bias and at least one of the predictors (see Tables 1, 2) and, as such, none of the other variables were included as controls. And based on research suggesting that there may be important age-related changes in the nature of a default negative valence bias across the adult lifespan, ultimately, age was included as an interactive effect with negative affect rather than a control (see below).

### Statistical analyses

Data were analyzed using Mplus^86^. Because of missing data for some surveys across different experiments, some scores were missing. Covariance coverage for the model ranged from 0.137 to 0.979, but a large majority (> 80%) exceeded 0.40. Table 3 reports sample size for each variable and Supplementary Table S2 reports covariance coverage. Further, full information maximum likelihood estimation (FIML^87^) is considered an optimal approach to addressing missing data patterns of this magnitude. Thus, by implementing FIML, we capitalized on all available data from this large dataset. Additionally, robust standard errors were estimated using the MLR estimator in Mplus to address any violations of univariate or multivariate normality. We applied a latent moderated structural equations method^57^ for testing moderation hypotheses. Multiple indices were used to assess global model fit. The comparative fit index (CFI > 0.90), Tucker–Lewis Index (TLI > 0.90), root-mean-square error of approximation (RMSEA < 0.08), and standardized root-mean residual (SRMR < 0.08) are reported. Once a model was determined to adequately fit the data, parameter estimates were interpreted. Three latent variables were modeled. The metric of the latent variables was set by fixing the variance of the latent variables to 1.00,thus, latent scores were standardized. First, scores from the BDI, DERS, NEON, STAIS, and STAIT were modeled as indicators of a latent variable representing negative affect. Second, scores from the EQ, IRQ, and NEOE were modeled as indicators of a latent variable representing social connectedness. Third, valence bias for faces (VB-faces and scenes) (VB-scenes) were modeled as indicators of a latent variable representing valence bias.

**Table 3:** Descriptive statistics of observed variables.

Variable	n	Mean (SD)	Sample Range	Possible Range
1. BDI	568	8.060 (7.182)	0–45	0–48
2. DERS	768	78.586 (24.019)	36–171	36–180
3. NEON	986	21.265 (8.996)	0–48	0–48
4. STAIS	1124	35.941 (11.027)	20–77	20–80
5. STAIT	1112	38.339 (11.738)	20–78	20–80
6. EQ	928	31.257	2–54	0–56
7. IRQ	651	77.725	16–112	16–112
8. NEOE	986	30.043	0–48	0–48
9. VB (Faces)	1361	63.593 (25.722)	0–100	0–100
10. VB (Scenes)	1145	47.501 (16.125)	4–100	0–100
11. Age	1277	28.063 (12.074)	17–88	

*BDI* Beck Depression Inventory, *DERS* Difficulties in Emotion Regulation Scale, N*E*ON Neuroticism, *STAIS* State Anxiety, *STAIT*-Trait Anxiety, *EQ* Empathy Quotient, *IRQ* Interpersonal Regulation Questionnaire, *NEOE* Extraversion, *VB* Valence Bias-higher scores associated with a more negative bias.

In this model, the latent scores of negative affect and social connectedness were used to predict valence bias. In addition, we explored interactive effects of age and negative affect, predicting that age might play a more critical role in the relationship between negative affect and valence bias given prior work demonstrating that increasing age is associated with a more positive valence bias^43,62^. In other words, we expected that the association between negative affect and valence bias would vary as a function of age such that negative affect might have a stronger impact on valence bias in older age when a more positive bias might otherwise be expected. In contrast, we did not have predictions about the effects of social connectedness as a function of age. Thus, we added the interaction between negative affect and age as a predictor of valence bias.

## Supplementary Information

Supplementary Information
