# Supplementary material for: Social connectedness and negative affect uniquely explain individual differences in response to emotional ambiguity
Source: Sci Rep. 2021 Feb 16;11:3870. doi: 10.1038/s41598-020-80471-2 (PMC7886913; doi:10.1038/s41598-020-80471-2)
Supplement: Supplementary file 1 — Supplementary Information [file 41598_2020_80471_MOESM1_ESM.docx]

Social connectedness and negative affect uniquely explain

individual differences in response to emotional ambiguity

*Maital Neta and Rebecca L. Brock

Department of Psychology

University of Nebraska-Lincoln

Correspondence concerning this article should be addressed to Maital Neta, mneta2@unl.edu.

**SUPPLEMENTARY TABLE 1:** Fourteen tasks combined in this dataset, including subtle methodological differences that were considered as potential control variables.

| Study Name | N | Stimulus presentation duration  (in ms) | Total # face trials | Total # IAPS trials | Response type | Location | Publication |
| --- | --- | --- | --- | --- | --- | --- | --- |
| Delay | 38 | 500 | 48 | 48 | button | lab | Neta & Tong (2016) |
| TimePerspective | 113 | 500 | 48 | 0 | button | lab | Neta, Tong, & Henley (2018) |
| ER | 82 | 500 | 48 | 0 | button | lab | Neta, Tong, Brown, Dunne, Clinchard, & Davis (under review) |
| ER-PsychoPhys | 87 | 500 | 48 | 48 | button | lab |  |
| Stress | 52 | 500 | 64 | 0 | mouse | lab | Brown, Raio, & Neta, 2017 |
| TimeDeadline | 24 | 500 | 48 | 0 | mouse | lab | Unpublished |
| Exercise | 576 | Indefinite | 48 | 48 | button | online | Neta, Harp, Beckford, Henley, & Koehler (2019) |
| Aging-YA | 55 | 500 | 48 | 48 | button | lab | Petro, Tong, Henley, & Neta (2018) |
| Aging-YA (new design) | 98 | 1000 | 48 | 48 | mouse | lab | Unpublished |
| Aging-OA | 44 | 500 | 48 | 48 | button | lab | Petro, Basyouni, & Neta (2020a) |
| Longitudinal | 79 | 500 | 48 | 48 | mouse | lab | Unpublished |
| MBSR | 4 | 1000 | 48 | 48 | mouse | lab | Unpublished |
| Exercise | 1 | 500 | 48 | 48 | mouse | lab | Unpublished |
| WorkingMemory | 137 | 500 | 48 | 48 | mouse | lab | Unpublished |
| *Note:* ER = Emotion Regulation, YA = Young Adults, OA = Older Adults, MBSR = Mindfulness-Based Stress Reduction | | | | | | | |

**SUPPLEMENTARY TABLE 2:** Covariance coverage of data.

| Variable | 1 | 2 | 3 | 4 | 5 | 6 | 7 | 8 | 9 | 10 | 11 |
| --- | --- | --- | --- | --- | --- | --- | --- | --- | --- | --- | --- |
| 1. BDI | .413 |  |  |  |  |  |  |  |  |  |  |
| 2. DERS | .192 | .576 |  |  |  |  |  |  |  |  |  |
| 3. NEON | .400 | .418 | .720 |  |  |  |  |  |  |  |  |
| 4. STAIS | .405 | .571 | .629 | .816 |  |  |  |  |  |  |  |
| 5. STAIT | .405 | .561 | .619 | .805 | .807 |  |  |  |  |  |  |
| 6. EQ | .355 | .418 | .673 | .584 | .575 | .674 |  |  |  |  |  |
| 7. IRQ | .137 | .443 | .350 | .500 | .500 | .350 | .502 |  |  |  |  |
| 8. NEOE | .400 | .418 | .720 | .629 | .619 | .673 | .350 | .720 |  |  |  |
| 9. VB (Faces) | .392 | .554 | .702 | .795 | .785 | .656 | .502 | .702 | .979 |  |  |
| 10. VB (Scenes) | .348 | .494 | .553 | .736 | .736 | .507 | .502 | .553 | .807 | .827 |  |
| 11. Age | .413 | .576 | .720 | .816 | .807 | .674 | .502 | .720 | .979 | .827 | 1.00 |
| *Note:* Percent of data present for each variable is reported along the diagonal, and covariance coverage is reported below the diagonal.  BDI = Beck Depression Inventory; DERS = Difficulties in Emotion Regulation Scale; NEON = Neuroticism; STAIS = State Anxiety; STAIT – Trait Anxiety; EQ = Empathy Quotient; IRQ = Interpersonal Regulation Questionnaire; NEOE = Extraversion; VB = Valence Bias – higher scores associated with a more negative bias | | | | | | | | | | | |

**SUPPLEMENTARY TABLE 3:** Unstandardized Model Results

|  | Unstandardized | |  |
| --- | --- | --- | --- |
|  | Estimate | SE | p-value |
| **Latent Variables** |  |  |  |
|  |  |  |  |
| Negative Affect |  |  |  |
| BDI | 6.18 | 0.338 | 0.000 |
| DERS | 18.28 | 0.726 | 0.000 |
| NEON | 7.58 | 0.240 | 0.000 |
| STAIS | 8.96 | 0.298 | 0.000 |
| STAIT | 11.20 | 0.281 | 0.000 |
|  |  |  |  |
| Social Connectedness |  |  |  |
| EQ | 5.54 | 0.469 | 0.000 |
| IRQ | 8.33 | 0.859 | 0.000 |
| NEOE | 7.15 | 0.418 | 0.000 |
|  |  |  |  |
| Valence Bias |  |  |  |
| VB-faces | 0.14 | 0.019 | 0.000 |
| VB-scenes | 0.06 | 0.007 | 0.000 |
|  |  |  |  |
| **Predictors of Valence Bias** |  |  |  |
|  |  |  |  |
| Social Connectedness | -0.28 | 0.097 | 0.004 |
| Negative Affect | 0.02 | 0.071 | 0.770 |
| Latent Interaction of Negative Affect and Age | 0.17 | 0.064 | 0.010 |
| Age (Standardized) | -0.22 | 0.069 | 0.001 |
|  |  |  |  |
| **Covariances** |  |  |  |
|  |  |  |  |
| Negative Affect – Social Connectedness | -0.52 | 0.040 | 0.000 |
| Negative Affect – Age | -0.17 | 0.030 | 0.000 |
| Social Connectedness – Age | -0.13 | 0.030 | 0.000 |
|  |  |  |  |

*Note:* BDI = Beck Depression Inventory; DERS = Difficulties in Emotion Regulation Scale; NEON = Neuroticism; STAIS = State Anxiety; STAIT – Trait Anxiety; EQ = Empathy Quotient; IRQ = Interpersonal Regulation Questionnaire; NEOE = Extraversion; VB = Valence Bias – higher scores associated with a more negative bias
